# Association between C-reactive protein and radiotherapy-related pain in a tri-racial/ethnic population of breast cancer patients: a prospective cohort study

**DOI:** 10.1186/s13058-019-1151-y

**Published:** 2019-05-28

**Authors:** Eunkyung Lee, Omar L. Nelson, Carolina Puyana, Cristiane Takita, Jean L. Wright, Wei Zhao, Isildinha M. Reis, Rick Y. Lin, WayWay M. Hlaing, Johnna L. Bakalar, George R. Yang, Jennifer J. Hu

**Affiliations:** 1 Department of Health Sciences, University of Central Florida College of Health Professions and Sciences, Orlando, FL 32816 USA; 2 Department of Public Health Sciences, University of Miami School of Medicine, Miami, FL 33136 USA; 3 Department of Radiation Oncology, University of Miami School of Medicine, Miami, FL USA; 4 Department of Radiation Oncology and Molecular Radiation Sciences, Johns Hopkins University, Baltimore, MD USA; 5 Sylvester Comprehensive Cancer Center, University of Miami School of Medicine, Miami, FL USA

**Keywords:** Breast cancer, Radiotherapy, Pain, C-reactive protein, Inflammatory biomarker

## Abstract

**Background:**

Post-surgery adjuvant radiotherapy (RT) significantly improves clinical outcomes in breast cancer patients; however, some patients develop cancer or treatment-related pain that negatively impacts quality of life. This study examined an inflammatory biomarker, C-reactive protein (CRP), in RT-related pain in breast cancer.

**Methods:**

During 2008 and 2014, breast cancer patients who underwent RT were prospectively evaluated for pre- and post-RT pain. Pre- and post-RT plasma CRP levels were measured using a highly sensitive CRP ELISA kit. Pain score was assessed as the mean of four pain severity items (i.e., pain at its worst, least, average, and now) from the Brief Pain Inventory. Pain scores of 4–10 were classified as clinically relevant pain. Multivariable logistic regression analyses were applied to ascertain the associations between CRP and RT-related pain.

**Results:**

In 366 breast cancer patients (235 Hispanic whites, 73 black/African Americans, and 58 non-Hispanic whites), 17% and 30% of patients reported pre- and post-RT pain, while 23% of patients had RT-related pain. Both pre- and post-RT pain scores differed significantly by race/ethnicity. In multivariable logistic regression analysis, RT-related pain was significantly associated with elevated pre-RT CRP (≥ 10 mg/L) alone (odds ratio (OR) = 2.44; 95% confidence interval (CI) = 1.02, 5.85); or combined with obesity (OR = 4.73; 95% CI = 1.41, 15.81) after adjustment for age and race/ethnicity.

**Conclusions:**

This is the first pilot study of CRP in RT-related pain, particularly in obese breast cancer patients. Future larger studies are warranted to validate our findings and help guide RT decision-making processes and targeted interventions.

**Electronic supplementary material:**

The online version of this article (10.1186/s13058-019-1151-y) contains supplementary material, which is available to authorized users.

## Background

Breast cancer is the most frequently diagnosed cancer and the second leading cause of cancer death among American women [1]. Compared to breast-conserving surgery (BCS) alone, adjuvant radiotherapy (RT) has significantly reduced loco-regional recurrences [2]. However, RT-induced adverse responses negatively impact patient overall quality of life (QOL). Breast erythema, pain, retraction at the tumor-bed site, fibrosis, cardiac morbidity, lymphedema, and telangiectasia are among the known adverse responses to RT [3–6]. Pain is one of the most prevalent symptoms and is an important QOL issue in breast cancer survivors [7–10].

A recent study reported the presence of racial-ethnic disparities in pain experience upon completion of RT [11], indicating the heterogeneity in the RT responses. The identification of a biomarker that can predict treatment-related symptoms is an important research question in the field of radiation oncology. Exposure to ionizing radiation induces immune/inflammatory responses to promote tissue repair [12], and elevated pro-inflammatory cytokines are potential biomarkers for RT-induced toxicities [13–15]. However, very few studies have examined biomarkers for RT-related pain. Recently, our lab reported that RT-induced skin toxicity was associated with an increase in plasma C-reactive protein (CRP) levels [15, 16]. This may suggest a potential relationship between inflammatory responses and RT-induced skin toxicities, which can be another source of treatment-related pain for patients with breast cancer.

CRP has been widely used as a robust inflammatory biomarker for many health conditions in both clinical and research settings, and several studies have shown a positive correlation between plasma CRP levels and pain intensity in cancer patients [17–19]; however, these results were from cross-sectional studies, which were often limited by uncertain temporal relationships or from the univariate analysis without adjustment for confounding variables [20, 21]. In addition, the study samples were limited to a specific racial/ethnic group, resulting in limited generalizability of the findings.

Therefore, we aimed to examine the associations between CRP levels and RT-related pain among breast cancer patients who underwent adjuvant RT using a prospective study design. We hypothesized that breast cancer patients with elevated CRP levels would be more likely to report pain, which may identify CRP as an inflammatory biomarker for pain. We also hypothesized that patients with elevated pre-RT CRP may be at higher risk in developing RT-related pain. Pain sensitization is one of the most important risk factors for persistent pain [22, 23]; thus, identifying potential biomarkers or mediators will be a critical strategy to identify those at risk of RT-related pain and targeted interventions among breast cancer patients.

## Methods

### Study design and patient population

Data for the current analysis was obtained from a prospective cohort study (University of Miami, FL, USA) where the goal was to examine the disparity of RT-induced early adverse skin reactions in a racially and ethnically diverse population of breast cancer patients. Briefly, the study recruited breast cancer patients from the Radiation Oncology clinics at the University of Miami Sylvester Comprehensive Cancer Center and Jackson Memorial Hospital in Miami, Florida, between December 2008 and August 2014. Patients were followed up for up to 12 months after the completion of RT. At the time of enrollment, each participant completed a self-administered baseline questionnaire. In addition, participants completed QOL questionnaire on the first day before initiation of RT, on the last day immediately after completion of RT, and at each follow-up visit (1, 2, 6, and 12 months). The current study only used QOL data collected on the first day of RT (i.e., pre-RT) and on the last day of RT (i.e. post-RT). The treating radiation oncologist met patients each week during the radiation treatment and evaluated adverse skin reactions at week 3 (mid-treatment), at week 6 (completion of RT), and at each follow-up visit. We collected blood samples (20 mL) at pre- and post-RT for biomarker data. Blood samples were processed within 2 h of phlebotomy, and the aliquoted plasma samples were stored at − 80 °C until assay. The study was approved by Institutional Review Boards of the University of Miami and Jackson Memorial Hospital, and all patients provided written informed consent.

The inclusion criteria were adult (≥ 18 years old at the time of diagnosis) female patients, newly diagnosed with breast cancer (AJCC stage 0–III) who had undergone BCS and planned to receive adjuvant RT to the whole breast with or without regional lymph nodes (total dose ≥ 40 Gy, dose per fraction ≥ 2.0 Gy). Other criteria included patients belonging to one of three racial/ethnic groups [self-reported non-Hispanic whites (NHW), black/African Americans (AA), and Hispanic whites (HW)] and being able to speak English or Spanish. The exclusion criteria were patients diagnosed with stage IV breast cancer and those that received partial breast irradiation and/or concurrent chemoradiation. Patients with missing pain score and/or CRP level at pre- or post-RT were excluded. To increase the validity of RT-related change in pain score, patients who reported pain due to other acute health conditions unrelated to cancer or radiation (such as shingles or fracture) were also excluded from the analysis after medical record verification.

### Radiation treatment

RT was delivered using standard or partially wide photon tangents using 6 and/or 10MV photons with forward planned field-in-field technique to maximize dose homogeneity. Patients received RT to the whole breast ± regional lymph nodes with conventional fractionation (2.0 Gy/day over 5–6 weeks, mostly 50 Gy in 25 fractions) or hypo-fractionation (> 2.0 Gy/day over 3 weeks, most commonly 42.4 Gy in 16 fractions). An additional boost dose of 10–20 Gy without bolus was delivered to the tumor-bed site in most patients. Radiation oncologists contoured target volumes, including the breast and lumpectomy cavity. The treatment plan was completed on the Eclipse or Pinnacle planning systems.

### Assessment of pain

All women enrolled in the study filled out the National Surgical Adjuvant Breast and Bowel Project (NSABP) B-39/RTOG 0413 protocol QOL questionnaire pre- and post-RT. This questionnaire measured QOL relating to breast cosmesis, fatigue, treatment-related symptoms, and perceived convenience of care. The section pertaining to treatment-related symptoms included four pain severity items, which were extracted from the Brief Pain Inventory (BPI): “Rate your pain at its worst, at its least, on average in the past four weeks, and now (0 = no pain to 10 = pain as bad as you can imagine).” The pain score was measured as a mean of these four pain severity items; a pain score of 4–10 was used to define the presence of clinically relevant pain because pain ≥ 4 indicates a moderate to severe level of pain, as used in previous studies [7, 24, 25]. In addition, patients who reported an increase in pain level from pre- to post-RT (i.e., pain score changed from < 4 to ≥ 4) was defined as having RT-related pain as previously reported [11] and compared to patients with pain score < 4 at both pre- and post-RT.

### Assessment of plasma CRP

Plasma CRP levels were measured using a high-sensitivity CRP enzyme-linked immunosorbent assay (ELISA) kit (Calbiotech, Spring Valley, CA) according to the manufacturer’s protocol, as previously described [16]. A standard curve was generated for each batch of samples based on CRP concentrations, which ranged from 0.2 to 10.0 mg/L. To ensure that the diluted samples were within the linear range of the standard curve, we re-ran the assays by adjusting the dilution ratio if samples were outside the detection range. The average coefficient of variation was 8.3%, and the inter-assay variation was less than 10%. The cut-off value of CRP level was determined based on clinical usage and literature review where CRP ≥ 10.0 mg/L is a prognostic biomarker for breast cancer survival [26]. For CRP change, we used 1.0 mg/L as the cut-off value because it has been significantly associated with RT-induced skin toxicity in the same patient population [16]. Considering that CRP is an acute-phase protein with a half-life of 18 h, we collected post-RT blood samples immediately after RT on the last day consistently among all sample patients.

### Assessment of covariates

Demographic information, self-reported race and ethnicity, comorbidities, and smoking history/status were obtained from a self-administered baseline questionnaire at the time of enrollment. A high correlation was found between the comorbidities reported on the questionnaires and those extracted from medical records [27, 28]. Tumor characteristics, such as tumor stage, estrogen receptor (ER), progesterone receptor (PR), human epidermal growth factor receptor 2 (HER2), and detailed information on treatments were ascertained from medical records.

### Statistical analysis

We first examined the distributions and frequencies of patient-, tumor-, and treatment-related characteristics overall and by race/ethnicity using the Pearson’s chi-square test or the Fisher’s exact test. The analysis of variance (ANOVA) was used to compare CRP levels by patient characteristics. The Pearson's chi-square test or the Fisher's exact test was used to compare the frequencies of elevated CRP or pain by patient characteristics. Univariable and multivariable logistic regression analyses were used to test whether elevated pre-RT CRP and/or obesity (BMI ≥ 30 kg/m^2^) were significantly associated with RT-related pain. Odds ratios (ORs) and 95% confidence intervals (95% CIs) were reported. In addition, we performed the receiver operating characteristics (ROC) curve analysis to evaluate whether pre-RT CRP level and/or obesity contribute to RT-related pain. A two-tailed *P* value < 0.05 was considered statistically significant, and all statistical analyses were performed using SAS 9.4 (SAS Institute, Cary, NC, USA).

## Results

### Patient population characteristics

The study population consisted of 366 breast cancer patients: 64% HW, 20% AA, and 16% NHW. The mean ± standard deviation (SD) of age was 56.0 ± 9.1 years. As shown in Table 1, AA women were more likely to have BMI ≥ 30 kg/m^2^, advanced stage or triple-negative tumors, larger volume (cc) of the breast, diabetes mellitus, and hypertension compared to HW or NHW women. HW women were more likely to receive hormone therapy (HT) with aromatase inhibitors prior to RT compared to other racial/ethnic groups. For breast cancer surgery, 68% of patients received BCS with or without sentinel lymph node biopsy (SLNB), and 32% received BCS with axillary lymph node dissection (ALND). For systemic therapy, about half of the patients received chemotherapy, 44% initiated HT prior to RT, and 7% began HT during RT. For RT, 84% of patients received conventional fractionation with a mean total dose of 58.2 ± 4.8 (SD) Gy, including an additional boost to the lumpectomy cavity, and 16% were treated with hypo-fractionated regimens. There were no significant differences in RT treatment regimens across the three racial/ethnic groups. Overall, patients reported a significantly higher pain score at post-RT (mean ± SD = 2.8 ± 2.5) compared to pre-RT (mean ± SD = 1.7 ± 2.1). In general, AA and HW patients had significantly higher pre-RT and post-RT pain scores compared to NHW patients.

**Table 1:** Patient demographic, tumor, and treatment characteristics by race/ethnicity

Variable	Categories	Total		NHW		AA		HW		*P* ^1^
		*N*	%	*N*	%	*N*	%	*N*	%	
Total		366	100	58	16	73	20	235	64	
Age (years)	< 50	95	26	18	31	19	26	58	25	0.613
	≥ 50	271	74	40	69	54	74	177	75	
	Mean (SD)	56.0 (9.1)		55.6 (9.1)		54.9 (9.2)		56.5 (9.1)		
BMI (kg/m^2^)	< 25	96	26	29	50	12	16	55	23	**<** *0.0001*
	25–29.9	124	34	16	28	17	23	91	39	
	≥ 30	146	40	13	22	44	60	89	38	
	Mean (SD)	29.3 (6.4)		26.6 (6.3)		32.6 (8.4)		28.9 (5.2)		
Smoking status	Never	240	66	37	64	51	70	152	64	0.490
	Former	107	29	20	34	17	23	70	30	
	Current	19	5	1	2	5	7	13	6	
Sum of 12 comorbid conditions^2^	0	147	40	28	48	19	26	100	43	0.119
	1	137	37	20	34	32	44	85	36	
	2	60	16	7	12	18	25	35	15	
	≥ 3	22	6	3	5	4	5	15	6	
Tumor stage	0	74	20	7	12	14	19	53	23	*0.003*
	IA-B	180	49	37	64	28	38	115	49	
	IIA-B	90	25	13	22	29	40	48	20	
	IIIA-C	22	6	1	2	2	3	19	8	
ER	Positive	279	76	43	74	49	67	187	80	0.072
	Negative	86	23	15	26	24	33	47	20	
PR	Positive	243	66	36	62	44	60	163	69	0.243
	Negative	122	33	22	38	29	40	71	30	
HER2	Positive	31	8	4	7	6	8	21	9	0.730
	Negative	275	75	50	86	56	77	169	72	
Triple negative	No	294	80	47	81	52	71	195	83	*0.005*
	Yes	54	15	8	14	20	27	26	11	
Axillary surgery	None/SLNB	248	68	39	67	54	74	155	66	0.439
	ALND	118	32	19	33	19	26	80	34	
Chemotherapy	No	195	53	31	53	39	53	125	53	0.999
	Yes	171	47	27	47	34	47	110	47	
Hormone therapy/initiation time	None/after RT	178	49	37	64	41	56	100	43	*0.015*
	Aromatase inhibitor before RT	98	27	9	16	14	19	75	32	
	Aromatase inhibitor during RT	14	4	3	5	2	3	9	4	
	Tamoxifen before RT	64	17	6	10	12	16	46	20	
	Tamoxifen during RT	12	3	3	5	4	5	5	2	
RT fractionation	Conventional	306	84	45	78	64	88	197	84	0.298
	Hypo	60	16	13	22	9	12	38	16	
Total RT dose (Gy)	< 60	107	29	21	36	18	25	68	29	0.348
	≥ 60	259	71	37	64	55	75	167	71	
	Mean (SD)	58.2 (4.8)		58.4 (4.6)		58.7 (4.9)		58.0 (4.8)		
Boost	Yes	331	90	56	97	65	89	210	89	0.225
	No	35	10	2	3	8	11	25	11	
Breast volume (cc)	< 892.1 (median)	183	50	38	66	20	27	125	53	*< 0.001*
	≥ 892.1 (median)	179	49	20	34	52	71	107	46	
	Mean (SD)	996 (532)		799 (464)		1254 (645)		965 (479)		
Pre-RT pain	Mean (SD)	1.7 (2.1)		1.0 (1.3)		2.0 (2.5)		1.8 (2.1)		*0.023*
Post-RT pain	Mean (SD)	2.8 (2.5)		1.9 (1.7)		3.2 (2.6)		2.8 (2.6)		*0.013*

^1^*P* values from the chi-square test or Fisher's exact test, or ANOVA, excluding missing. Significant findings are in italics

^2^Sum of 12 patient-reported comorbid conditions: diabetes, hypertension, heart disease, lung disease, thyroid disease, cirrhosis liver, stroke, chronic bronchitis, hepatitis, tuberculosis, and 2 others

*Abbreviations*: *NHW* non-Hispanic whites, *AA* black or African American, *HW* Hispanic whites, *SD* standard deviation, *BMI* body mass index, *ER* estrogen receptor, *PR* progesterone receptor, *HER2* human epidermal growth factor receptor 2, *SLNB* sentinel lymph node biopsy, *ALND* axillary lymph node dissection, *RT* radiotherapy

### Plasma CRP levels at pre- and post-RT and RT-related CRP change

As shown in Table 2, there was no significant difference between pre- (mean ± SD = 6.5 ± 9.3) and post-RT (mean ± SD = 6.1 ± 8.9) plasma CRP levels. The CRP levels were significantly higher in obese patients at both pre- and post-RT. Pre-RT CRP levels were significantly higher in patients with pre- or post-RT pain score ≥ 4. Post-RT CRP levels were significantly higher in patients with smoking history, post-RT pain score ≥ 4, larger breast volume, and tamoxifen treatment during RT.

**Table 2:** CRP levels by patient, treatment characteristics, and pain status

Variable	Pre-RT CRP (mg/L)					Post-RT CRP (mg/L)				
	*N*	Mean	SD	MD	*P* ^1^	*N*	Mean	SD	MD	*P* ^1^
Study population	362	6.5	9.3	3.5		338	6.1	8.9	3.5	0.646^2^
Race/ethnicity										
NHW	58	6.1	12.4	2.8	0.879	53	5.2	12.1	2.2	0.541
AA	71	6.9	8.0	4.4		66	7.0	6.7	5.5	
HW	233	6.4	8.8	3.5		219	6.0	8.6	3.7	
Age (years)										
< 50	94	6.2	10.7	3.1	0.797	86	5.6	10.1	2.8	0.571
≥ 50	268	6.5	8.8	3.7		252	6.2	8.5	3.9	
BMI (kg/m^2^)										
< 25	94	3.1	6.3	1.2	*0.0001*	87	3.1	8.4	1.4	*0.0009*
25–29.99	124	7.3	11.0	3.7		117	6.5	9.5	3.8	
≥ 30	144	8.0	8.8	4.9		134	7.7	8.4	5.3	
Smoking history										
Never	238	5.8	8.6	3.4	0.066	225	5.4	7.8	3.4	*0.046*
Ever	124	7.7	10.3	3.7		113	7.4	10.7	3.9	
Pre-RT pain score										
< 4	286	5.9	8.8	3.3	*0.014*	266	5.6	8.4	3.4	0.054
≥ 4	59	9.3	11.8	4.9		56	8.1	10.3	4.2	
Post-RT pain score										
< 4	230	5.4	8.2	3.1	*0.007*	226	5.3	7.8	3.2	*0.014*
≥ 4	101	8.3	9.9	4.6		99	7.9	11.0	4.8	
Tumor stage										
0	73	6.1	8.2	3.6	0.916	65	6.3	8.3	3.4	0.872
IA-B	180	6.4	10.0	3.3		165	6.3	10.8	3.4	
IIA-III	109	6.7	8.8	3.9		108	5.7	5.7	4.2	
Breast volume (cc)										
< 892.1 cc (median)	181	5.2	8.2	2.6	*0.011*	169	5.0	9.2	2.4	*0.021*
≥ 892.1 cc (median)	177	7.7	10.2	4.6		165	7.2	8.6	5.0	
Hormone therapy										
None/after RT	175	6.7	10.5	3.2	0.736	159	5.7	7.9	3.3	*0.032*
AI before	97	6.7	7.7	4.8		95	7.2	10.0	4.6	
AI during	14	4.5	4.0	3.3		14	5.1	5.3	3.8	
Tamoxifen before	64	5.5	8.8	2.8		59	4.3	6.0	2.4	
Tamoxifen during	12	8.2	9.3	5.1		11	12.8	21.0	5.1	
RT fractionation										
Conventional	302	6.4	9.1	3.5	0.632	289	6.2	9.2	3.5	0.575
Hypo	60	7.0	10.2	3.5		49	5.4	7.0	3.7	

^1^*P* values from ANOVA; significant findings are in italics

^2^Paired *t* test comparing pre- and post-RT CRP

*Abbreviations*: *NHW* non-Hispanic whites, *AA* black or African American, *HW* Hispanic whites, *BMI* body mass index, *AI* aromatase inhibitor, *SD* standard deviation, *MD* median

### Clinically relevant pain by selected variables and CRP levels

As shown in Table 3, the proportion of patients who reported clinically relevant pain (pain score ≥ 4) increased from 17% at pre-RT to 30% at post-RT. Pre-RT pain was more prevalent in patients with AA or HW race/ethnicity, BMI ≥ 30 kg/m^2^, HER2-positive tumor, received trastuzumab alone or taxane+trastuzumab, received ALND, or pre-RT CRP ≥ 10 mg/L, compared to their respective comparison groups. Post-RT pain was more prevalent in patients with AA or HW race/ethnicity, age < 50 years, BMI ≥ 30 kg/m^2^, at least 2 comorbid conditions, conventional RT fractionation, total RT dose ≥ 60 Gy, or pre-RT CRP ≥ 10 mg/L, compared to their respective counterparts. About 23% of patients had RT-related pain, and it was more frequent in patients with AA or HW race/ethnicity, at least 2 comorbid conditions, conventional RT fractionation, or RT-induced CRP change > 1 mg/L.

**Table 3:** Pre-RT, post-RT, and RT-related pain by selected variables and CRP status

Variable	Categories	Pre-RT pain^1^ (*N* = 349)					Post-RT pain^1^ (*N* = 335)					RT-related pain^2^ (*N* = 262)				
		No (< 4)		Yes (≥ 4)			No (< 4)		Yes (≥ 4)			No		Yes		
		*N*	%	*N*	%	*P* ^3^	*N*	%	*N*	%	*P* ^3^	*N*	%	*N*	%	*P* ^3^
Total		290	83	59	17		233	70	102	30		203	77	59	23	
Race/ethnicity	NHW	53	96	2	4	*0.016*	45	88	6	12	*0.003*	42	89	5	11	*0.018*
	AA	58	82	13	18		42	60	28	40		37	66	19	34	
	HW	179	80	44	20		146	68	68	32		124	78	35	22	
Age (years)	< 50	75	81	17	19	0.639	51	60	34	40	*0.027*	46	69	21	31	0.045
	≥ 50	215	84	42	16		182	73	68	27		157	81	38	19	
BMI (kg/m^2^)	< 25	84	88	11	12	*0.009*	68	81	16	19	*0.001*	63	85	11	15	0.075
	25–29.99	101	88	14	12		85	74	30	26		73	78	20	22	
	≥ 30	105	75	34	25		80	59	56	41		67	71	28	29	
Sum of 12 comorbid conditions^4^	0	114	84	22	16	0.897	103	75	34	25	*0.009*	87	83	18	17	*0.009*
	1	111	83	22	17		88	73	33	27		79	81	18	19	
	2	48	83	10	17		32	57	24	43		28	64	16	36	
	≥ 3	17	77	5	23		10	48	11	52		9	56	7	44	
HER2	Positive	18	62	11	38	*0.004*	16	59	11	41	0.294	10	71	4	29	0.676
	Negative	220	84	42	16		177	69	79	31		155	76	48	24	
Chemotherapy	None	159	85	27	15	0.140	128	72	50	28	0.455	115	80	29	20	0.416
	Taxane	123	79	32	21		100	68	48	32		83	75	27	25	
	Other	8	100	0	0		5	56	4	44		5	63	3	38	
Trastuzumab	No	274	85	49	15	*0.005*	218	70	92	30	0.281	194	78	55	22	0.497
	Yes	16	61	10	39		15	60	10	40		9	69	4	31	
Taxane+trastuzumab	None/other chemo only	166	86	26	14	*0.012*	132	71	53	29	0.558	120	79	31	21	0.667
	Either	109	82	24	18		87	68	40	32		74	75	25	25	
	Both	15	62	9	38		14	61	9	39		9	75	3	25	
Axillary surgery	None/SLNB	205	86	33	14	*0.027*	163	72	63	28	0.141	145	78	40	22	0.590
	ALND	85	77	26	23		70	64	39	36		58	75	19	25	
RT fractionation	Conventional	240	82	52	18	0.309	190	67	94	33	*0.013*	165	75	55	25	*0.028*
	Hypo	50	88	7	12		43	84	8	16		38	90	4	10	
Total RT dose (Gy)	< 60	92	89	11	11	*0.045*	74	80	19	20	*0.014*	66	84	13	16	0.123
	≥ 60	198	80	48	20		159	66	83	34		137	75	46	25	
Pre-RT CRP (mg/L)	< 10	256	85	45	15	*0.006*	210	73	79	27	*0.001*	183	79	48	21	0.056
	≥ 10	30	68	14	32		20	48	22	52		17	63	10	37	
Post-RT CRP (mg/L)	< 10	234	83	47	17	0.410	203	71	82	29	0.077	175	78	49	22	0.373
	≥ 10	32	78	9	22		23	58	17	43		22	71	9	29	
RT-related CRP change (mg/L)	≤ 1	192	82	41	18	0.992	170	72	67	28	0.140	151	82	34	18	*0.006*
	> 1	70	82	15	18		53	63	31	37		43	65	23	35	

^1^Pain score ≥ 4 (moderate or severe pain) was considered yes for clinically relevant pain

^2^Patients with pre-RT pain score < 4 and post-RT pain score ≥ 4 or < 4 were considered yes or no for RT-related pain

^3^*P* values were from the chi-square test or Fisher's exact test excluding missing. Significant findings are in italics

^4^Sum of 12 patient-reported comorbid conditions: diabetes, hypertension, heart disease, lung disease, thyroid disease, cirrhosis liver, stroke, chronic bronchitis, hepatitis, tuberculosis, and 2 others

### Plasma CRP levels by pain status

In Table 4, we summarize CRP levels in 4 or 8 groups of patients and identified significantly higher CRP levels (mean ± SD = 10.8 ± 12.1) in 34 patients with pain scores ≥ 4 at both pre- and post-RT. We have also identified 20 patients with pain score ≥ 4 at pre-RT but < 4 at post-RT. In stratified analysis by obesity, we identified 11 non-obese patients with high pre-RT CRP also had pain scores ≥ 4 at both pre- and post-RT. Therefore, we limited subsequent data analysis of RT-related pain to only two groups of patients with pre-RT pain score <  4 and post-RT score either < 4 (no) or ≥ 4 (yes).

**Table 4:** CRP levels by pre- and post-RT pain stratified by obesity

BMI	Pre-RT pain	Post RT pain	*N*	Pre-RT CRP				Post-RT CRP				
				Mean	SD	Median	*P* ^1^	Mean	SD	Median	*P* ^1^	*P* ^2^
NA	No	No	194	5.5	8.4	3.3	0.278	5.2	7.9	3.2	*0.034*	0.675
NA	No	Yes	57	7.1	8.8	3.4		7.2	10.8	4.8		0.936
NA	Yes	No	20	5.4	9.2	2.2	*0.010*	5.6	9.5	3.3	0.075	0.807
NA	Yes	Yes	34	10.8	12.1	6.0		8.8	10.2	5.6		0.278
< 30	No	No	130	5.1	9.2	2.6	0.786	4.4	8.1	2.0	0.169	0.423
< 30	No	Yes	31	5.4	8.1	2.7		7.4	14.1	3.2		0.366
< 30	Yes	No	10	4.4	7.0	2.2	0.393	3.6	2.4	3.1	0.647	0.676
< 30	Yes	Yes	11	12.1	16.8	3.6		7.5	11.5	4.2		0.304
≥ 30	No	No	64	6.3	6.3	4.6	0.480	6.9	7.1	4.8	0.368	0.497
≥ 30	No	Yes	26	9.0	9.5	5.9		6.9	4.8	5.9		0.259
≥ 30	Yes	No	10	6.4	11.3	2.7	*0.022*	7.7	13.3	3.5	0.142	0.114
≥ 30	Yes	Yes	23	10.1	9.4	6.4		9.4	9.7	6.9		0.677

^1^Unadjusted *P* value from the Wilcoxon two-sample test (comparing 2 groups by pain status)

^2^*P* value from the paired *t* test within each group (comparing pre-RT and post-RT CRP). Significant findings are in italics

### Association between pre-RT CRP and RT-related pain

In Table 5, we evaluated the association of elevated pre-RT CRP (≥ 10 mg/L) and/or obesity with RT-related pain. In multivariable model, there was a significant association between high pre-RT CRP and RT-related pain (OR = 2.44, 95% CI = 1.02, 5.85) regardless of obesity status. In obese patients, there was a stronger association between high pre-RT CRP and RT-related pain (OR = 3.71, 95% CI = 1.05, 13.09) than in non-obese patients (OR = 1.36, 95% CI = 0.35, 5.39). Therefore, we conducted a combined analysis to show that patients with BMI ≥ 30 kg/m^2^ and pre-RT CRP ≥ 10 mg/L had 4.73-fold elevated risk for RT-related pain (95% CI = 1.41, 15.81) compared to patients with BMI <  30 kg/m^2^ and pre-RT CRP < 10 mg/L. All models were adjusted for age and race/ethnicity.

**Table 5:** Association between pre-RT CRP and RT-related pain by obesity

BMI	Pre-RT CRP	*N*	%	RT-related pain		Univariable		Multivariable^1^	
				*N*	%	OR (95%CI)	*P*	OR (95%CI)	*P*
< 30	NA	161	64	31	54	Ref		Ref	
≥ 30	NA	90	36	26	46	1.70 (0.93, 3.11)	0.082	1.49 (0.80, 2.78)	0.211
NA	< 10 mg/L	225	90	47	82	Ref		Ref	
NA	≥ 10 mg/L	26	10	10	18	*2.37 (1.01, 5.55)*	*0.048*	*2.44 (1.02, 5.85)*	*0.046*
< 30	< 10 mg/L	148	92	28	90	Ref		Ref	
< 30	≥ 10 mg/L	13	8	3	10	1.29 (0.33, 4.98)	0.716	1.36 (0.35, 5.39)	0.659
≥ 30	< 10 mg/L	77	86	19	73	Ref		Ref	
≥ 30	≥ 10 mg/L	13	14	7	27	*3.56 (1.07, 11.91)*	*0.039*	*3.71 (1.05, 13.09)*	*0.041*
< 30	< 10 mg/L	148	59	28	49	Ref		Ref	
< 30	≥ 10 mg/L	13	5	3	5	1.29 (0.33, 4.98)	0.716	1.34 (0.34, 5.26)	0.678
≥ 30	< 10 mg/L	77	31	19	33	1.40 (0.73, 2.72)	0.315	1.22 (0.62, 2.42)	0.567
≥ 30	≥ 10 mg/L	13	5	7	12	*5.00 (1.56, 16.03)*	*0.007*	*4.73 (1.41, 15.81)*	*0.012*

^1^All models were adjusted for age (< 50, ≥ 50) and race/ethnicity (NHW, HW, AA). Significant findings are in italics

We also present ROC curves of high pre-RT CRP and/or obesity in predicting RT-related pain for (A) all, (B) NHW, (C) HW, and (D) AA patients and their corresponding area under the curve (AUC). The gray line represents the theoretical performance of the variable equivalent to a coin toss. The blue line is for obesity (BMI ≥ 30 kg/m^2^), the red line is for pre-RT CRP ≥ 10 mg/L, and the green line shows the combined effect of obesity and pre-RT CRP ≥ 10 mg/L. The results show some improvements of AUC in the combined BMI and pre-RT CRP model for NHW (AUC = 0.6540) and AA (AUC = 0.6524) patients (see Additional file 1: Figure S1).

## Discussion

Postoperative adjuvant RT significantly reduces local-regional recurrence and improves breast cancer survival. Therefore, there has been increasing usage of adjuvant RT in early-stage breast cancer patients. However, RT is associated with skin toxicities and other late effects that negatively impact QOL. We evaluated whether the inflammatory biomarker, CRP, was associated with RT-related pain. To the best of our knowledge, this is the first study to date reporting a significant association between pre-RT CRP and RT-related pain.

Consistent with literature, the proportion of patients who experienced clinically relevant pain increased from pre-RT (17%) to post-RT (30%) [7, 29]. Pre-RT pain may be related to other cancer treatments (e.g., surgery and/or chemotherapy). Intriguingly, a higher proportion of patients with at least two comorbid conditions showed an elevated risk for post-RT pain [30]. It is notable that not all patients reported an increase in pain score after RT. Specifically, 194 patients reported pain score < 4 at both pre- and post-RT. A total of 57 patients reported the change of pain score from < 4 at pre-RT to ≥ 4 at post-RT. Twenty patients reported pain score change from ≥ 4 at pre-RT to < 4 at post-RT. Thirty-four patients reported pain score ≥ 4 at both pre- and post-RT. These findings are consistent with another study among breast cancer patients, which reported that cancer pain was not static, but rather could progress or regress [25]. Inter-individual variations in pain may be related to differences in responses to RT, genetic factors, and inflammatory responses.

The CRP level in normal human serum ranges from 0.2 to 10 mg/L; 90% of apparently healthy individuals have CRP levels < 3 mg/L; and only 1% have levels ≥ 10 mg/L. In our study, 13% and 13% of patients had pre-RT and post-RT CRP ≥ 10 mg/L, respectively (Table 3). Radiation sensitivity is a complex and inherited polygenic trait, with many genes in multiple biological pathways. Genetic studies are warranted to elucidate the contribution of genetic variants in racial/ethnic differences of RT-related pain. In addition, a higher proportion of AA patients were obese (60%), compared to 22% of NHW and 38% of HW patients, respectively. Other studies have also reported that a higher proportion of AA women had elevated inflammatory cytokines including CRP and interleukin (IL)-6, relative to NHW women [31, 32]. This may explain, in part, why AA patients experience more cancer treatment-related symptoms such as pain, skin toxicity, nausea/vomiting, and depression compared to NHW patients [11, 33–35].

Multiple studies have shown that irradiation increases immune/inflammatory responses [12, 36], and there is evidence showing a positive correlation between elevated inflammatory cytokines and pain severity in both human [17, 19] and animal studies [37, 38]. In addition to pain, elevated pro-inflammatory cytokines, including CRP, after cancer treatment have been associated with persistent fatigue and sleep disturbances in breast cancer patients [18, 39]. These findings may suggest the existence of a shared etiology in cancer treatment-related symptoms. Given that immune/inflammation underscores cancer treatment-related symptoms, the use of anti-inflammatory agents as prophylactic treatment may be considered.

Our current data provides evidence that CRP is associated with RT-related pain in breast cancer patients. Our findings have several clinical implications. First, elevated plasma CRP has been associated with cancer prognosis, vascular atherosclerosis, insulin resistance, and type 2 diabetes mellitus that may impact overall survival. Therefore, patients with elevated post-RT CRP levels should be actively monitored for other medical conditions that may also impact overall survival. Second, considering the involvement of CRP in fatigue and prognosis of breast cancer, future follow-up studies will focus on monitoring CRP levels, QOL, and clinical outcomes. Third, growing evidence suggests that plasma CRP is positively associated with sugar intake but negatively associated with dietary intakes of minerals, vitamins, and polyunsaturated fatty acids [40]. Therefore, modulating CRP concentrations by modifying dietary intakes may be a promising intervention strategy. Lastly, we observed a stronger association between elevated pre-RT CRP and RT-related pain in obese patients. Considering that CRP and BMI are highly correlated, weight reduction may also reduce pre-RT CRP levels and RT-related pain.

Multiple studies have shown the predictive value of CRP in cancer outcomes [41–43]. This study further adds to the literature by reporting a significant association between elevated pre-RT CRP level and RT-related pain. However, using a threshold AUC of 0.8 by ROC analysis, combining BMI and pre-RT CRP levels may not be a strong predictor for RT-related pain. With a limited sample size, we did not include many other clinical or treatment variables. Larger studies are warranted to further test our predictive models, which should include other patient/clinical variables and additional promising biomarkers to improve their utilities in predicting RT-related pain.

There are several strengths and limitations of this study. First, we used a prospective study design that is particularly suitable to conduct biomarker research and RT-related pain. We followed patients and collected biological samples over time and recorded patient-reported QOL on the first and last day of RT to minimize recall bias, which provides more precise estimates of biomarkers and pain. This is the first study showing racial/ethnic differences in pre- and post-RT pain, which may help bridge the knowledge gap regarding the mechanisms of racial/ethnic disparities in cancer treatment-related QOL.

Several limitations should also be taken into consideration. First, because CRP is a non-specific inflammatory biomarker, CRP levels can be influenced by multiple factors including anti-inflammatory drug use and/or other health conditions. Second, despite the prospective cohort study design, some covariates (i.e., comorbidities) were collected only one point in time. The lack of repeated measures prevented us from capturing changes in health status, which may influence CRP and pain levels. Third, some variables that may influence individual patient’s pain experience and CRP level (i.e., the use of pain medication and anti-inflammatory agents) were not available for this study, thus should be considered for future studies. Fourth, the nature of pain (nociceptive or neuropathic) may be differently influenced by inflammatory responses; however, the detailed pain quality data was not available in the current analysis. Lastly, we used patient-reported information on comorbid conditions, which might introduce reporting bias. However, many studies have reported high reliability of self-reported information when compared to medical records [27, 28].

## Conclusions

In summary, our current data show a significant association between elevated pre-RT CRP and RT-related pain in breast cancer patients. More importantly, we demonstrate for the first time that obese patients with pre-RT CRP ≥ 10 mg/L have a significantly increased risk of RT-related pain compared to non-obese patients with pre-RT CRP < 10 mg/L. Therefore, our current data suggest that there is an association between inflammatory responses and RT-related pain. Our results will need to be validated externally in other study populations. If validated, these results pave the way for testing anti-inflammatory agents in reducing RT-related pain.

## Additional file

Additional file 1: **Figure S1.** ROC curves analysis of high pre-RT CRP and/or obesity in RT-related pain. (A) All, (B) NHW, (C) HW, and (D) AA patients and their corresponding AUC for RT-related pain. The grey solid line represents the theoretical performance of the variable equivalent to a coin toss. The blue line represents obesity (BMI≥30), the red line represents pre-RT CRP ≥10 mg/L, and the green line presents the combined effect of obesity and pre-RT CRP ≥ 10 mg/L. (PDF 57 kb)

## Abbreviations

AA: African American/black
ALND: Axillary lymph node dissection
BCS: Breast-conserving surgery
BMI: Body mass index
CI: Confidence interval
CRP: C-reactive protein
ER: Estrogen receptor
HER2: Human epidermal growth factor receptor 2
HT: Hormone therapy
HW: Hispanic whites
NHW: Non-Hispanic whites
OR: Odds ratio
PR: Progesterone receptor
QOL: Quality of life
RT: Radiotherapy
SD: Standard deviation
SLNB: Sentinel lymph node biopsy
