# Supplementary figures and images for: Association between C-reactive protein and radiotherapy-related pain in a tri-racial/ethnic population of breast cancer patients: a prospective cohort study
Source: Breast Cancer Res. 2019 May 28;21:70. doi: 10.1186/s13058-019-1151-y (PMC6537305; doi:10.1186/s13058-019-1151-y)

**(A) All (N=251)**

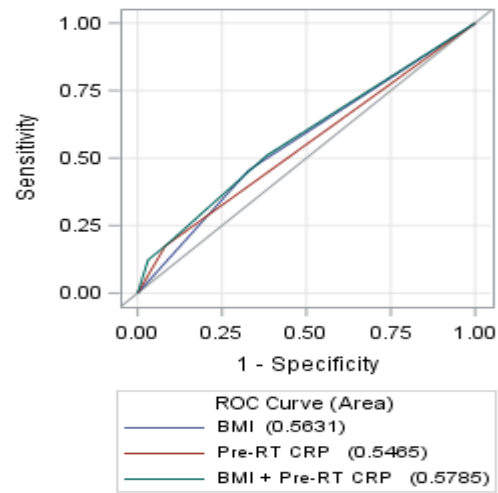

**(B) NHW (N=51)**

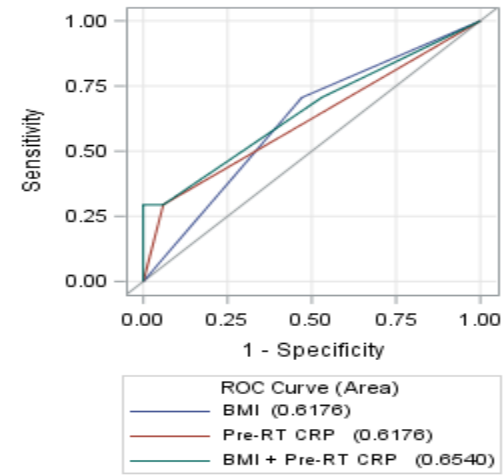

**(C) HW (n=153)**

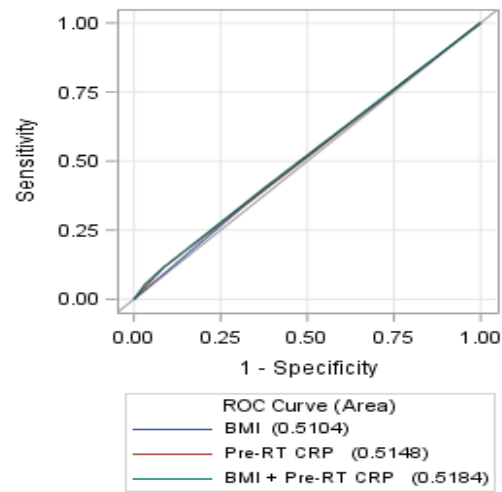

**(D) African Americans (N=47)**

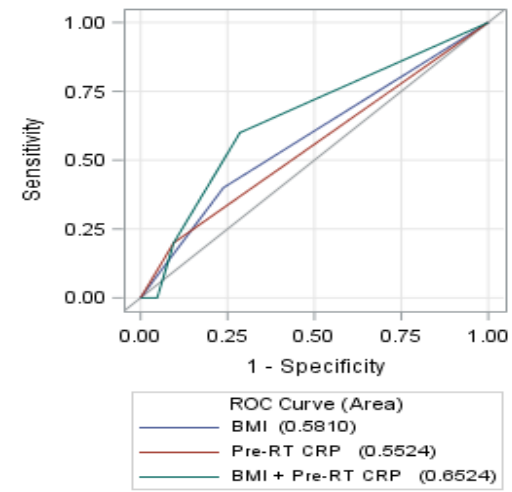

Supplement: Supplementary file 1 — Figure S1. ROC curves analysis of high pre-RT CRP and/or obesity in RT-related pain. (A) All, (B) NHW, (C) HW, and (D) AA patients and their corresponding AUC for RT-related pain. The grey solid line represents the theoretical performance of the variable equivalent to a coin toss. The blue line represents obesity (BMI≥30), the red line represents pre-RT CRP ≥10 mg/L, and the green line presents the combined effect of obesity and pre-RT CRP ≥ 10 mg/L. (PDF 57 kb) [file 13058_2019_1151_MOESM1_ESM.pdf]
